# Cardiovascular Magnetic Resonance and Sport Cardiology: a Growing Role in Clinical Dilemmas

**DOI:** 10.1007/s12265-020-10022-7

**Published:** 2020-05-20

**Authors:** Viviana Maestrini, Camilla Torlasco, Rebecca Hughes, James C. Moon

**Affiliations:** 1 Department of Cardiovascular, Respiratory, Nephrology, Anesthesiology and Geriatric Sciences, Sapienza University of Rome, Viale del Policlinico 155, 00161 Rome, Italy; 2 Department of Medicine and Surgery, University of Milano-Bicocca, Milan, Italy; 3 Department of Cardiovascular, Neural and Metabolic Sciences, S.Luca Hospital, Istituto Auxologico Italiano, IRCCS, Milan, Italy; 4 Institute of Cardiovascular Science, University College London, Gower Street, London, UK; 5 Barts Heart Centre, Advanced Cardiac Imaging and The Inherited Cardiovascular Diseases Unit, St Bartholomew’s Hospital, West Smithfield, London, EC1A 7BE UK

**Keywords:** Athlete’s heart, CMR, Sport cardiology

## Abstract

Exercise training induces morphological and functional cardiovascular adaptation known as the “athlete’s heart” with changes including dilatation, hypertrophy, and increased stroke volume. These changes may overlap with pathological appearances. Distinguishing athletic cardiac remodelling from cardiomyopathy is important and is a frequent medical dilemma. Cardiac magnetic resonance (CMR) has a role in clinical care as it can refine discrimination of health from a disease where ECG and echocardiography alone have left or generated uncertainty. CMR can more precisely assess cardiac structure and function as well as characterise the myocardium detecting key changes including myocardial scar and diffuse fibrosis. In this review, we will review the role of CMR in sports cardiology.

## CMR and Athletes

Prolonged and intense exercise training induces morphological and functional cardiovascular adaptation known as the “athlete’s heart.” Changes include left ventricular hypertrophy (LVH), left ventricular (LV) and right ventricular (RV) cavity dilatation and increased stroke volume. Intense prolonged training can induce changes that overlap with cardiomyopathy [1]. Distinguishing athletic cardiac remodelling from cardiomyopathy is important. Underlying cardiac disease can be promoted by intensive physical activity, whilst adrenergic surge, dehydration and electrolyte imbalances may trigger malignant ventricular arrhythmia. The medical dilemma is frequently between risking a preventable catastrophic event (by not disqualifying an athlete from competitive sport) versus un-necessary disqualification, with often profound lifestyle and livelihood impact.

Combined with non-imaging data (especially the electrocardiogram), transthoracic echocardiography (TTE) is the first line test due to low cost and wide availability. Cardiac magnetic resonance (CMR) is increasingly used as it can refine discrimination of health from a disease where echocardiography alone has left or generated uncertainty [2, 3]. CMR has advantages for structure/functional assessment combined with tissue characterisation. Sports cardiology can also call upon additional diagnostic strategies in complex cases such as exercise assessment, cascade screening, re-evaluation after detraining or genotyping. CMR can help gatekeeper such strategies or remove their need altogether. CMR, as with r any other structure/functional assessment, cannot diagnose pure channelopathies, but one advantage is that it can definitively state that structure/function is normal, increasing certainty.

## CMR Overview

**Fig. 1:**
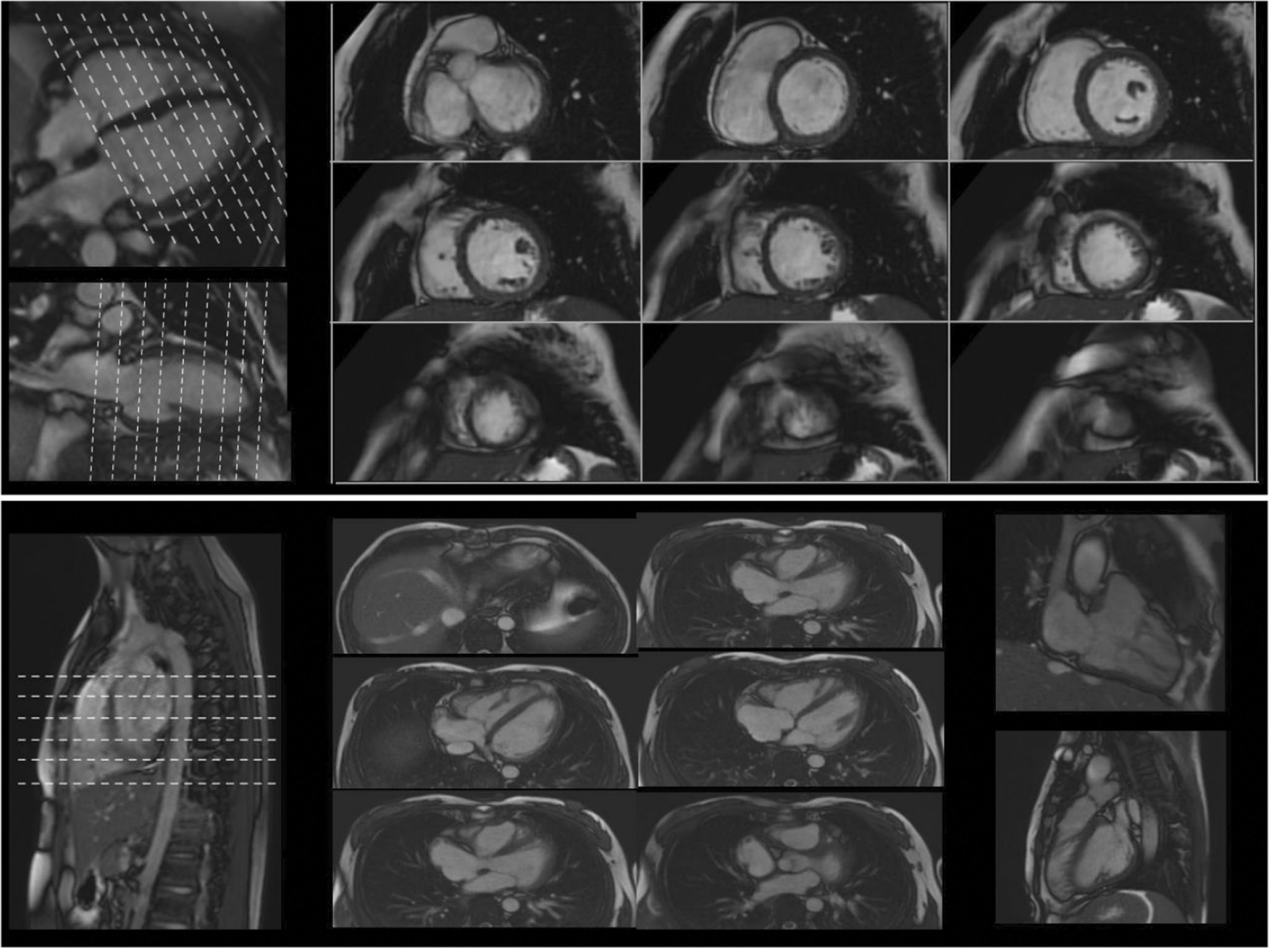
Biventricular chambers, size, function and mass by CMR. In the top panel, a short axis stack covers both ventricles from the atrioventricular valve plane to the apex, and planned on 4- and 2-chamber views. The short axis stack is then used to accurately calculate mass, volumes and function by contouring endocardial and epicardial borders. The bottom panel shows a transverse RV stack, an RV 2-chamber and outflow tract view used for RV regional wall motion and morphology

**Fig. 2:**
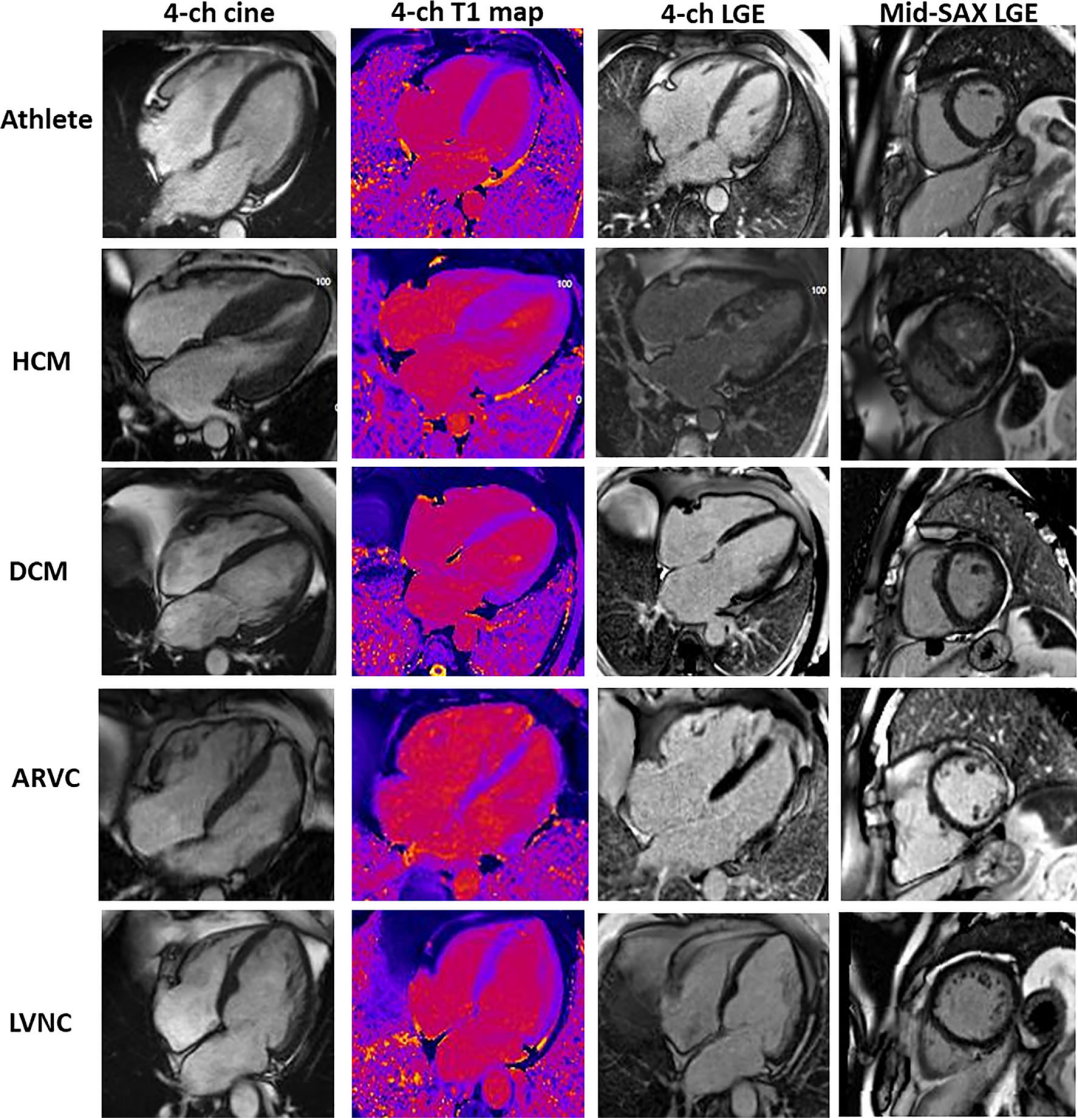
CMR findings in the athlete’s heart and mimics. Images show (left to right) 1) an end-diastolic 4-chamber cine; 2) a 4-chamber T1 map; 3) a 4-chamber LGE; 4) a mid-ventricular short axis LGE; in the following conditions (top to bottom): **a** athlete: demonstrating enlarged cardiac chambers with normal wall thickness. Native T1 map and 4-chamber LGE view are normal. There is inferior RV insertion point LGE, a normal finding when it is just a gram or so, as here. **b** HCM: predominantly septal LVH. Patchy elevated native T1 with patchy mid-wall LGE seen in hypertrophied myocardium and (here) the papillary muscles. **c** DCM: LV dilatation with normal native T1 map and no LGE. **d** ARVC: biventricular ARVC. Elevated native T1 is appreciated within the RV wall as well as extensive biventricular LGE. **e** LVNC: non-compaction of the LV. Native T1 map is normal and there is subtle LGE seen in the 4-chamber image within the apical cap. This appearance has to be interpreted with context, particularly of ethnicity and athleticism

CMR is the gold standard for heart chamber morphology and wall motion assessment, for size and mass calculation and for biventricular systolic function quantification, which are performed with fewer geometrical assumptions compared to echocardiography and with no blind spots (the apex, the basal anterior wall—areas where early LVH manifests in hypertrophic cardiomyopathy—, the inferior RV insertion point) (Fig. 1). Multiparametric tissue characterisation, particularly with the use of gadolinum-based contrast agent (GBCA) may detect disease (especially for sports cardiology, focal ischemic or non-ischemic scar) missed by structure and function alone, helping differentiate between athleticism and early stage cardiomyopathy (Fig. 2) [3]. The principle technique by CMR for tissue characterisation is late gadolinium enhancement imaging but T1, T2 and extracellular volume mapping add value by measuring myocardial composition changes that may link to detectable disease [4].

Native (precontrast) T1 is increased weakly by the presence of fibrosis and strongly by amyloid and oedema. T1 falls with fatty infiltration and iron overload [5–8]. In the absence of a contrast agent (or iron), native T2 is only known to increase—this occurs with oedema [9] for example during acute myocarditis. When combined with gadolinium-based contrast administration, T1 mapping allows the calculation of extracellular volume fraction (ECV), i.e. permits the relative volumes of the extra- and intracellular myocardial compartments to be quantified [10]. Generally expressed as a percentage of myocardial mass, ECV is a marker of myocardial tissue remodelling and provides a physiologically intuitive unit of measurement. Apart from acute oedema and amyloid, its increase is usually due to excessive collagen deposition and is thus a robust measure of diffuse myocardial fibrosis, as observed, for example in HCM. Remarkable in athletic LVH, ECV falls (as does native T1 slightly) suggesting that physiological athletic LVH represents disproportionate cellular hypertrophy, unlike any other sort of LVH assessed to date [11].

Finally, late gadolinium enhancement (LGE) imaging post contrast administration is used to identify focal myocardial fibrosis (MF). Broadly, MF can be divided into ischaemic (looks like an infarct: always involving the sub-endocardium, often territorial, may be transmural) and non-ischaemic (mid-myocardium or subepicardial). The pattern of LGE provides information on aetiology, whilst the extent may indicate risk—risk of heart failure and risk of sudden death. With high resolution imaging, some LGE can be physiological (for example small amounts at the RV insertion points) [12]. In athletes, RV insertion point LGE and LGE in the trabeculae is more common than that in sedentary patients, whilst in competitive male veteran athletes, other forms of scar are observed including ischaemic pattern (up to 10% of subjects) where it is associated with different coronary plaque morphology compared with the general population [13]. These may be from type 2 myocardial infarction via demand ischaemia, but the significance remains controversial because whilst this sort of scar associates with risk markers, its interaction with the global benefit of exercise is unknown, leaving the management of individual subjects with such scar unclear [13, 14].

## Athlete’s Heart Versus Cardiomyopathy

The human heart senses demand and adapts both short and long term [15]. The long-term changes are well known to include symmetrical four chamber dilatation together with different degrees of LVH which may overlap with cardiomyopathy features, for example many athletes will have RV sizes that exceed major criteria for ARVC. Some of the challenges on the evaluation of the athletic remodelling of the heart are the measurement of the training load—especially in amateurs—and the difficulties to obtain reference ranges sport and sex specific [16, 17]—as well as the additional impact of ethnicity or performance enhancing drug use [3, 18]. An additional compounding factor is that many genetic predispositions require a “second hit” with overlap and causality being partly predisposition, partly training.

**Fig. 3:**
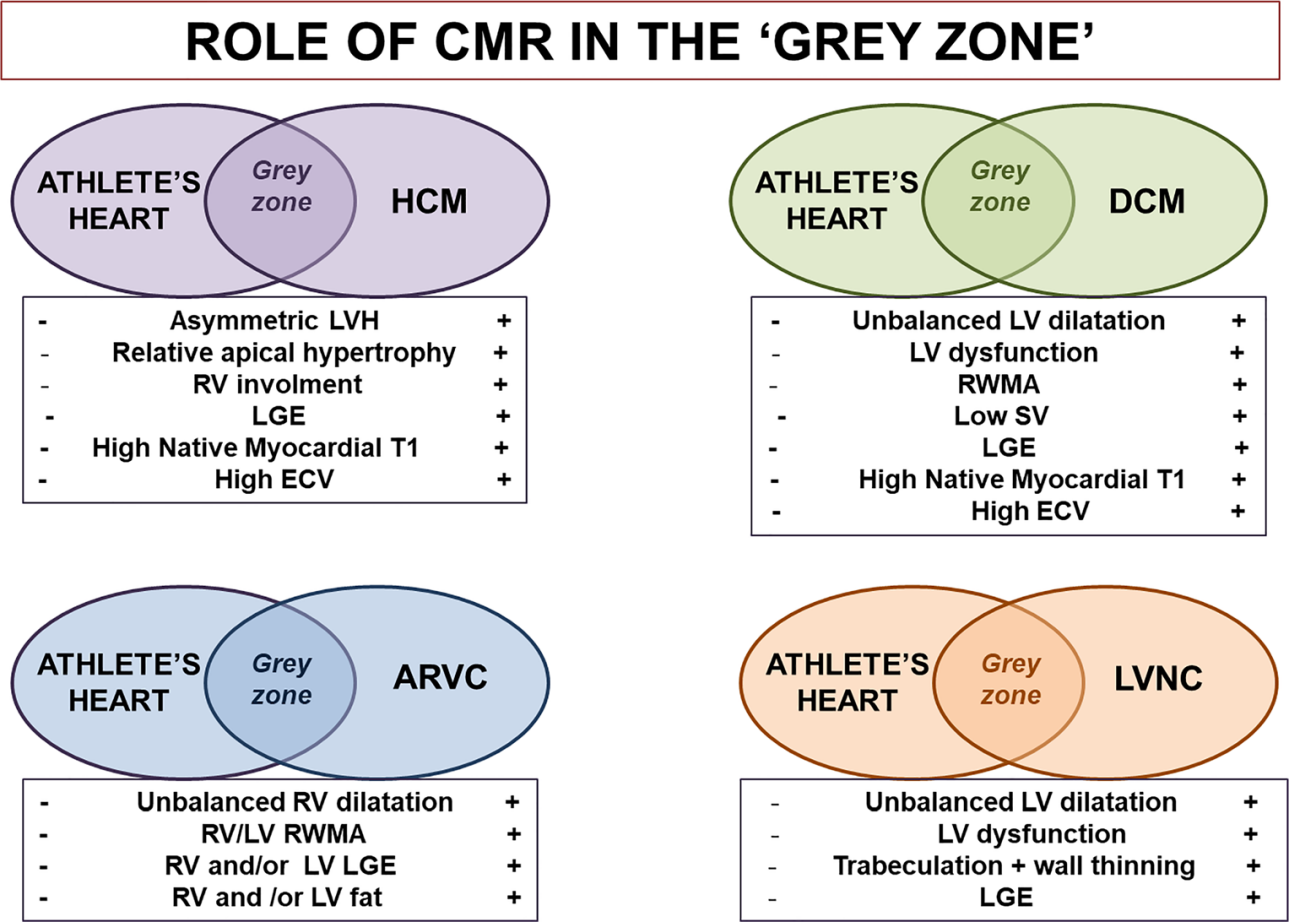
Role of CMR in the grey zone. The figure shows the role of CMR in the grey zone between athlete’s heart and cardiomyopathy. For every scenario, CMR features are shown. ARVD: arrhythmogenic right ventricular cardiomyopathy; DCM: dilated cardiomyopathy; ECV: extracellular volume fraction; HCM: hypertrophic cardiomyopathy; LGE: late gadolinium ehancement; LV: left ventricle; LVH: left ventricle hypertrophy; LVNC: left ventricular non-compaction; RV: right ventricle; SV: stroke volume; RWMA: regional wall motion abnormalities

CMR is helpful because measurement precision is superior. Relative features can be helpful, for example disproportionate RV or LV dilatation can point to ARVC or DCM, whilst disproportionate hypertrophy, especially if focal can point to HCM (Fig. 3). Morphological variation can generate small areas of apparent thinning (clefts for example), but scar away from the trabeculae or RV insertion points which implies a past or ongoing cardiomyopathic process.

## Hypertrophic Cardiomyopathy

**Fig. 4:**
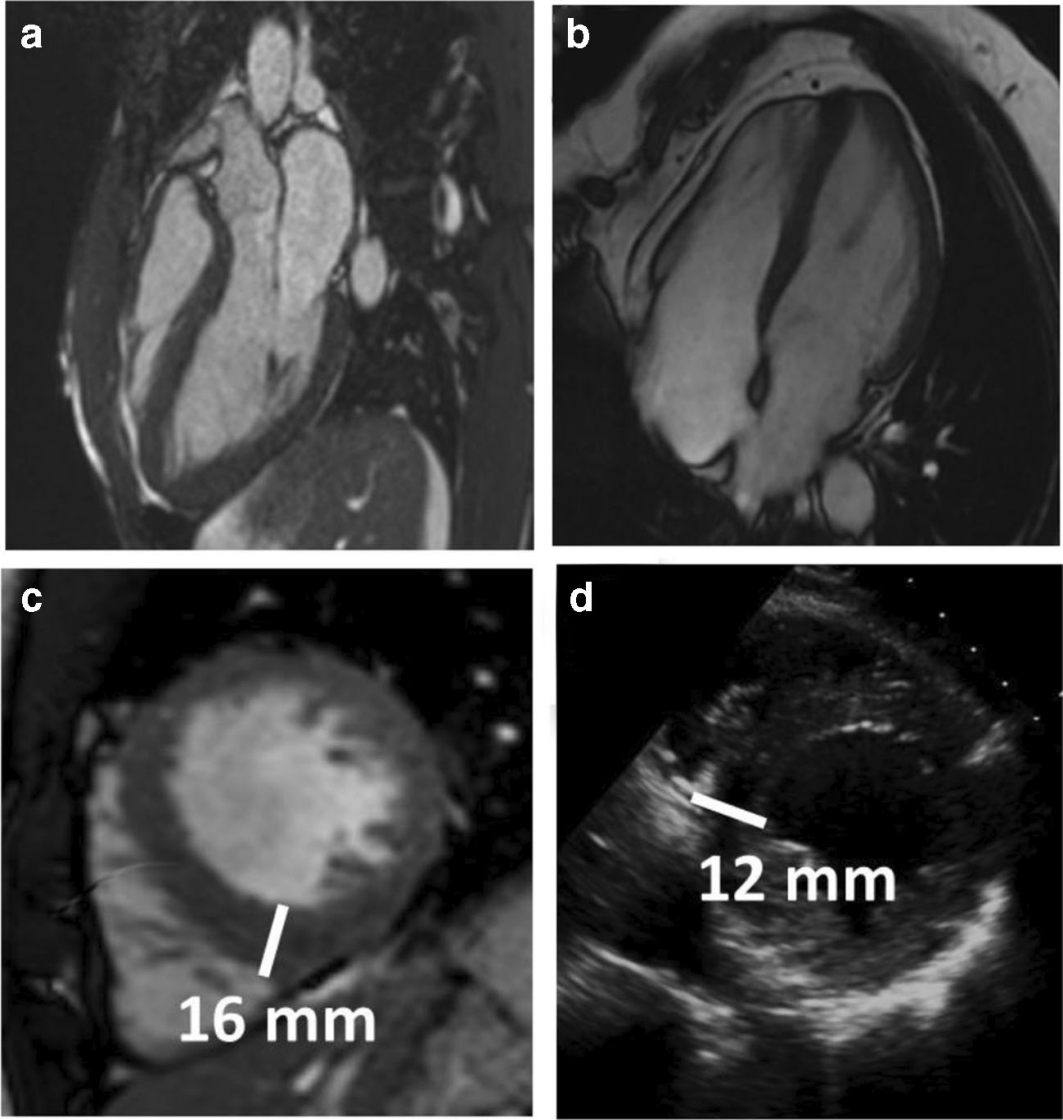
Focal hypertrophy potentially missed by echocardiography. Two athletes presenting with focal hypertrophy limited to the apex (**a**, **b**). **c** Short axis view by CMR showing focal hypertrophy limited to the inferior septum with a maximum wall thickness (16 mm) measured at the RV insertion point in an athlete. **d** The corresponding short-axis view by echocardiography underestimated the hypertrophy (12 mm)

**Fig. 5:**
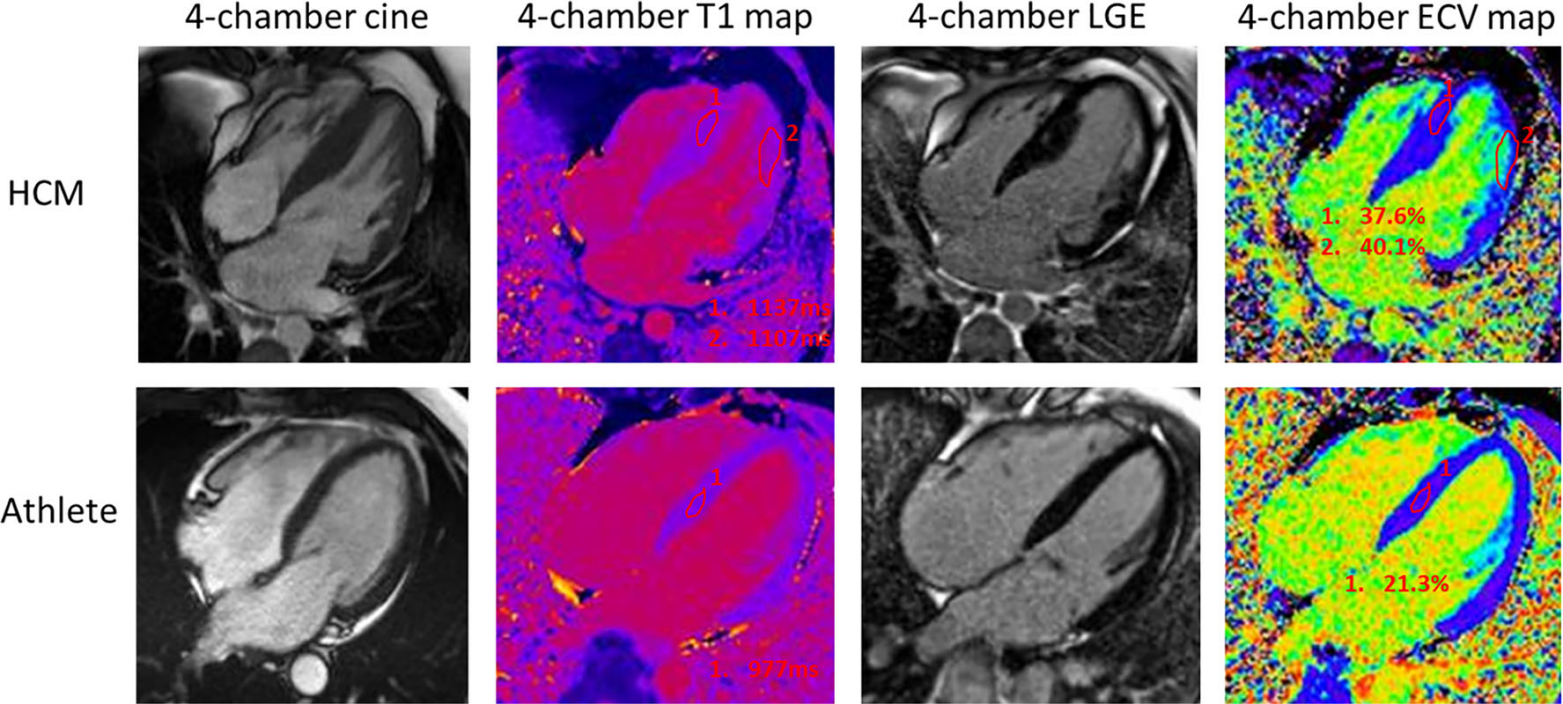
Multiparametric CMR imaging comparing HCM with the athlete’s heart. End diastolic 4-chamber cine of a patient with HCM showing septal thickening. Two regions of interest are drawn within the myocardium on native T1 mapping with elevated native T1 values (1107–1137 ms, range 950–1060 ms on 1.5 T Siemens Aera) with corresponding LGE and elevated ECV (37.6–40.1%, range 24–28% on 1.5 T Siemens Aera). In comparison, an athlete’s heart demonstrates normal wall thickness on 4-chamber cine with normal native T1 values in the thickest region (basal septum; 977 ms), no LGE and low ECV (21.3%)

One of the most common and challenging differential diagnoses is distinguishing between physiological left ventricular hypertrophy (LVH) induced by sporting activity and mild phenotypic expression of hypertrophic cardiomyopathy (HCM). In athletes, LVH can reach 15 mm; however, this was described in male rowers and is always associated with increased LV volumes [19, 20]. Echocardiography can miss focal hypertrophy in subjects with poor acoustic windows, particularly when hypertrophy affects the apex, the basal antero-lateral wall or the inferior septum or inferior septum [21], while CMR has the ability to explore every segments (Fig. 4) including the RV [22]. CMR should be requested in case of pathological ECG changes, particularly abnormal T wave inversion—according with age and ethnicity—with normal echocardiogram that can identify prephenotypic variant as relative apical HCM (extensive T wave inversion on ECG and relative but not absolute apical hypertrophy) [23]. Furthermore, CMR can reveal features of cardiomyopathy that are non-diagnostic per sè, but are known to be present in phenotype-negative/genotype-positive HCM individuals, such as papillary muscle abnormalities [24], basal-apical muscle bundle [25] and clefts [26]. The presence of LGE, particularly in the more hypertrophied myocardium, shifts the diagnosis away from the athlete’s heart, in favour of HCM. Recently, the introduction of the mapping technique provided new features. In HCM, extracellular matrix expansion and myocardial disarray lead to a high ECV while in athletes, increasing LV mass is associated with the fitness level and is secondary to intracellular expansion (ECV becomes relatively smaller) [11]. Preliminary results on a small cohort showed the potential use of mapping to distinguish between the athlete’s heart (low ECV) and HCM (high ECV) [27] (Fig. 5) [28]. Perfusion mapping can reveal microvascular dysfunction also in the absence of LVH and LGE [29]. In some selected cases, a detraining period may demonstrate partial LVH regression, a process characterised by ECV returning to normal via intracellular regression [30].

## Dilated Cardiomyopathy

Dilated cardiomyopathy is a heterogeneous disease characterised by the presence of left ventricular or biventricular dilatation and systolic dysfunction in the absence of abnormal loading conditions or coronary artery disease sufficient to cause global systolic impairment [31–33]. Several non-genetic causes have been identified and inflammatory cardiomyopathies are increasingly recognised. A positive family history is prevalent in DCM patients (up to 60% of cases) [34], and a genetic cause can be identified in up to 40% of familial DCM [35]. The two most frequently involved genes are titin (*TTN*, 20–25% of all familiar DCM cases) and lamin A/C (*LMNA*, 5–10%). The latter, along with cardiac sodium channel NAv1.5 (*SCN5A*), filamin C (*FLNC*) and desmoplakin (*DSP*) mutations, is associated with a more aggressive disease and an arrhythmogenic phenotype [36]. This ‘arrhythmogenic DCM’ can occur in up to one third of DCM patients and overlaps with the current concept of arrhythmogenic cardiomyopathy [37].

DCM has a long preclinical stage, in which patients are mostly asymptomatic and present with minor cardiac abnormalities, followed by an overt phase, characterised by systolic heart failure-like manifestations [32]. Ventricular arrhythmias, including those causing sudden cardiac death, can occur at any stage. One third of DCM patients present non-ischaemic scar on LGE images at diagnosis, often septal mid-wall, which is associated with poorer prognosis [38]. Nevertheless, the absence of LGE does not exclude the diagnosis.

The athlete’s heart shares features with DCM. Dose-dependent exercise-induced cardiac chamber enlargement and mildly reduced ejection fraction (EF) at rest are common in competitive athletes, especially in endurance sports. However, in athletes, this is associated with bradycardia and high stroke volume with balanced RV dilatation [39]. In unclear cases, stress echocardiography may help. Athletes are able to increase their cardiac output, SV and EF during exercise [40], patients with DCM less so [41]. Recent experiences with in-scanner exercise real-time CMR protocols appear promising in differentiating between physiology and pathology, although further research is needed [42, 43]. Finally, parametric mapping and ECV provide useful tools in differentiating adaptation to exercise from DCM. T1 mapping, T2 mapping and ECV have all been demonstrated to be lower in athletes than in DCM patients, although T1 mapping appeared the most accurate in order to differentiate the two conditions [44].

## Arrhythmogenic Right Ventricular Cardiomyopathy

Arrhythmogenic right ventricular cardiomyopathy (ARVC) is a genetically inherited heart muscle disease, predominantly associated with mutations in the genes encoding desmosomal proteins and with an estimated prevalence of 1 in 2000–5000 people [45]. It is characterised by progressive fibro-fatty replacement of myocardium and malignant ventricular arrhythmias [46]. The clinical spectrum is wide with gene-dependent LV and biventricular variants [47–49].

A correct and timely diagnose of ARVC in athletes can be critical and challenging. Physical exercise can promote disease expression and progression, triggering malignant arrhythmias [50]. Endurance sports are associated with younger age at presentation and SCD—up to 20% of SCD in athletes are related to ARVC [51, 52]. The mechanism for exercise-induced ARVC is thought to be via repetitive wall stress [53] that may promote the breakdown of the desmosome that eventually triggers fibro-fatty replacement of the RV walls and results in the phenotype, acting as a second hit [54–57].

However, many conditions can resemble ARVC, posing a diagnostic dilemma [58]. Heart displacement may alter the ventricular repolarisation ECG patterns and distort the right ventricle. RV overload (e.g. intracardiac shunts, pulmonary hypertension) may dilate and alter RV morphology, whilst there may be other scarring pathologies that affect the RV inflammatory cardiomyopathy [59] and sarcoidosis [60]. To add complexity, athletic adaption itself share some features with ARVC, so that the traditional diagnostic criteria may lead to overdiagnosis when applied to athletes. ECG abnormalities, e.g. anterior T wave inversion [61] and RVOT ventricular ectopic can be observed in healthy athletes. Furthermore, a significant proportion of athletes exhibit dimensional and morphological changes in the RV, consistent with ARVC, including RV outflow tract dilatation (30–40%) and rounded RV apex (more than 80%) [62].

In asymptomatic athletes with a negative family history, balanced biventricular dilatation is likely to be benign but a RV ejection fraction < 45% on CMR, RV end-diastolic volume/LV end-diastolic volume > 1.3:1, regional wall motion abnormalities in the RV and the presence of typical LV/RV LGE are highly indicative of ARVC [63]. Perhaps more than any disease, ARVC diagnosis requires multiple lines of evidence, with ECG screening remaining important [51] while CMR is used in cases where the ECG is pathological. Nevertheless, ARVC in athletes remains a challenging diagnosis, and all the screening tool are limited by the wide phenotype spectrum, which can include a long subclinical phase and SCD as the first manifestation.

## Left Ventricular Non-Compaction

Originally considered rare, left ventricular non-compaction (LVNC) is increasingly identified by imaging. It is an inherited cardiomyopathy characterised by a thin, compacted epicardial layer and an extensive, non-compacted endocardial layer with increased LV trabeculation and trabecular recesses [64]. The prognosis of rare, overt LVNC is poor [65] but there is no shared consensus on diagnostic criteria, and LVNC is being over-diagnosed in athletes, particularly black athletes [64, 65]. The remodelled athletes’ heart has 12–20% greater LV wall thickness and 10% larger biventricular cavity sizes [66] increasing ascertainment and the volume of trabeculae. One observational study of 1000 athletes diagnosed 8.1% as having LVNC using Chin and Jenni criteria compared with 7% of 415 sedentary controls; however, only 0.9% of these athletes had abnormal resting indices or ECG changes that would warrant the diagnosis [64]. Another study of 2500 Olympic athletes found that 1.4% had excessive trabeculations suggestive of LVNC yet when considering other features such as arrhythmias, only 0.1% were likely to have a true diagnosis [66].

True LVNC has a variable clinical presentation, and non-compaction may be a component of other cardiomyopathies. Symptoms include palpitations, chest pain, heart failure and rarely aborted/sudden cardiac death or thromboembolism [67]. Athletes with LVNC more commonly present with brief syncope, whereas athletes with physiologic adaptation mimicking LVNC are asymptomatic [65], but these associations are clouded by diagnostic uncertainty.

The electrocardiogram (ECG) in LVNC is abnormal in up to 87% of cases but variable, with no definitive criteria. The most common ECG finding in athletes with LVNC is LVH by voltage criteria [68], with other arrhythmias detected on Holter or stress testing, all non-specific findings. LGE if present is supportive of myopathy and relates to adverse clinical features, but both athletes and LVNC may have no LGE so sensitivity is low [69].

Increased LV trabeculation is found in up to 30% of patients with LV systolic dysfunction of any cause [70]. Cardiac adaption can increase the actual or apparent degree of trabeculation—for example in pregnancy [71]. Athletic adaption therefore risks causing increased ascertainment and over-diagnosis, but special attention is still warranted to those athletes who demonstrate other diagnostic findings. Newer criteria such as fractal analysis with ethnically appropriate reference ranges are likely helpful. Advice on sports participation in athletes reaching LVNC criteria is given on a per-patient basis and relates to symptoms, degree of myocardial dysfunction and extent of trabeculations, with the risk of over-diagnosis being given high priority compared perhaps to other diseases [9].

## Athlete’s with Ventricular Arrhythmias

Ventricular arrhythmias can be a warning sign of an underlying cardiac condition. CMR can reveal abnormal myocardial tissue usually missed by echocardiography; however, there are no definitive studies exploring the role of LGE as a prognostic tool in athletes. The data are limited to a small study where 72 athletes presenting with complex ventricular arrhythmias and non-ischemic LGE had a worse prognosis [72] and an ever smaller study confirmed the not benign meaning of LGE [73].

## Veteran Athlete’s Heart

It is indisputable that regular aerobic exercise is beneficial for primary and secondary prevention of cardiovascular disease; however, the impact of lifelong endurance exercise on the heart has only recently been explored. In the minority of susceptible veteran athletes, exercise can precipitate adverse events like sudden cardiac death (SCD), which in two thirds of cases, is attributable to silent coronary artery disease (CAD) [74]. Several studies have demonstrated a higher than the expected prevalence of CAD in veteran athletes [13, 75], and a failure to detect those at risk using routine cardiovascular screening such as exercise testing has potentially devastating consequences. Cardiac CT including calcium scoring has shown CAD in 25–53% veteran athletes and has prognostic benefits of determining plaque morphology and total atherosclerotic burden.

There is an ongoing debate on the possible negative effect of lifelong sporting activity on the heart. CMR plays an important role in assessing the veteran athlete’s heart. Physiological adaptation includes a lack of LGE and lower myocardial native T1 and ECV than controls. When comparing veteran athletes with controls, LGE CMR imaging showed a significantly higher scar burden; overt myocardial scar was detected in 11.4% vs 0% controls, mainly in males (15.2 vs 2.2%) [14]. Association has been not proven at this stage and further prospective studies need to be performed. Other studies have similarly identified non-ischaemic scar as a solely male athlete phenomenon (17% of male athletes vs 0% controls) and have demonstrated a link between scar and higher systolic blood pressure at peak exercise and higher LV mass index, suggesting that myocardial fibrosis is related to exercise-induced hypertension and myocardial hypertrophy [12]. The same study also demonstrated a link between the level of endurance activity (i.e. distances swam/cycled in triathletes) and LGE, which could easily be extrapolated to the veteran athletes when considering the cumulative effect of exercise in this group.

It appears that the impact of long-term athleticism on the veteran athlete’s heart exhibits significant gender variation and more work is required to understand why male athletes demonstrate more pathological findings than women [76]. Also, the long-term impact of differing exercise disciplines on the heart (i.e. endurance vs resistance exercise) would be interesting to explore.

## Conclusion

CMR requests in the sports cardiology field are growing. CMR provides substantial certainty in many ‘grey area’ cases. It can integrate anatomy, function and tissue characterisation in a single imaging test and has particular utility when athletes present with an abnormal electrocardiogram or ventricular arrhythmias and either apparently normal echocardiography or echocardiographic changes that may or may not relate to athletic adaptation. Particular findings of utility are the presence of hypertrophy, RV involvement and significant scar. CMR needs however to be performed appropriately [2] with appropriate reference ranges for male and particularly for female athletes, and in the context of the different sporting categories and training regimens. Caution should be exercised to avoid over-interpreting normal findings such as minor RV insertion point LGE or the extent of trabeculae.
